# Food insecurity and hypertension prevalence, awareness, and control in the Eastern Caribbean Health Outcomes Research Network Study

**DOI:** 10.1371/journal.pgph.0003296

**Published:** 2025-05-05

**Authors:** Carol R. Oladele, Neha Khandpur, Deron Galusha, Sanya Nair, Saria Hassan, Vivien Wambugu, Josefa Martinez-Brockman, Marcella Nunez-Smith, Rafael Perez-Escamilla

**Affiliations:** 1 Equity Research and Innovation Center, Yale School of Medicine, New Haven, Connecticut, United States of America; 2 Wageningen University and Research, Wageningen, The Netherlands; 3 Emory University School of Medicine, Emory Rollins School of Public Health, Atlanta, Georgia, United States of America; 4 Department of Social and Behavioral Sciences, Yale School of Public Health, New Haven, Connecticut, United States of America; New York University, UNITED STATES OF AMERICA

## Abstract

Limited evidence exists on the association between food insecurity (FI) and the hypertension care cascade in the Caribbean despite the high burden of both. The objective of this study is to examine the relationship between FI and hypertension prevalence, awareness, and control in the Eastern Caribbean. We conducted a cross-sectional analysis of baseline data (2013–2018) from the Eastern Caribbean Health Outcomes Research Network Cohort Study (n = 2961). Food insecurity was measured using the Latin American and Caribbean Food Security Scale (ELCSA) and classified as secure, mild, moderate, and severe. Hypertension was defined using guidelines from the Seventh Report of the Joint National Committee on Prevention and Caribbean Health Research Council. Poisson regression was used to estimate prevalence ratios for the association between FI and hypertension prevalence, awareness, and control, adjusting for covariates. Overall prevalence of FI was 28 percent in our sample. Seventeen percent experienced mild, 6 percent moderate, and 4 percent experienced severe FI. Fifty-eight percent had hypertension, 65 percent were aware of their hypertension, and among those aware, 56 percent had uncontrolled hypertension. Model results showed no association between FI and hypertension prevalence and awareness. Results for control showed moderate FI (PR = 1.33, CI = 1.09–1.64) and severe FI (PR = 1.30, CI = 1.05–1.62) were associated with 30 percent higher prevalence of uncontrolled hypertension compared to those who are food secure. Sex-stratified results showed women with moderate (PR = 1.39, CI = 1.13–1.71) and severe FI (PR = 1.41, CI = 1.16–1.72) had 40 and 41 percent higher prevalence of uncontrolled hypertension compared to food secure women, respectively. Results for men were not statistically significant. Findings align with prior evidence of greater FI prevalence among women and associations with hypertension control. Nutrition policies are needed to reduce FI prevalence and increase access to affordable, nutritious foods. Results warrant further studies to understand sex differences in FI prevalence and the impact on hypertension.

## Introduction

Food insecurity (FI) is defined by the World Health Organization (WHO) as when all people at all times have physical, social, and economic access to sufficient, safe, nutritious food that meets their dietary needs and food preferences [1]. Estimates from the Food and Agriculture Organization of the United Nations (FAO) show that 2.3 billion people (29%) experienced moderate or severe food insecurity in 2021 [2]. Evidence also shows a widening gender disparity in the prevalence of FI [2]. FI has increased during the last ten years, with much of the increase stemming from the COVID-19 pandemic. An estimated 41 percent of Caribbean households experienced food insecurity in 2020, an increase from 32 percent pre-pandemic [3]. There has also been a 38 percent increase in the number of people who reported going without food for an entire day between 2021 and 2022 [4]. FI is associated with cardiovascular risk factors, including hypertension [5,6]. In the Caribbean region, hypertension prevalence ranges 18–38 percent and is a leading cause of morbidity and a primary risk factor for heart disease and stroke, both leading causes of death in the Caribbean [7,8].

While findings are mixed, some evidence shows FI is positively associated with hypertension and poor hypertension control. Studies show FI is associated with higher systolic blood pressure and higher odds of hypertension compared to those without FI, independent of demographic, economic, and lifestyle risk factors [5,9–11]. This positive association has been demonstrated across population groups and varied methods of assessing hypertension. Studies also show that individuals with FI are less likely to achieve hypertension control and adhere to medication regimens compared to those who are food secure [10,12]. Women are also more likely to experience FI and related negative health consequences compared to men. Though fewer studies focus on hypertension compared to other outcomes, findings are consistent with evidence for outcomes like diabetes and obesity that demonstrate negative health impacts of FI. Most of the existing evidence, however, is concentrated in the US and other areas within the Americas. Limited research has been conducted in the Caribbean, leading to significant knowledge gaps in the region.

Several factors have been identified as important contributors to FI in the Caribbean. These include strong reliance on imported food, poverty, and climate change impacts [13]. Countries in this region import 60–90 percent of their food [14–16]. The high reliance on imported food contributes to FI as these foods tend to be expensive, have limited nutritional value, and are oftentimes ultra-processed. Prior work in the Eastern Caribbean showed affordability as a main determinant of fruit and vegetable consumption, primarily affecting individuals who experienced FI [17]. Increasing poverty due to weak economic markets and joblessness has also been correlated with higher prevalence of FI [18]. Extreme weather events due to climate change have affected food production and availability in the region, leading to increased reliance on food imports [13]. These interrelated factors have wide-ranging consequences, including poor dietary quality—a major risk factor for diet-sensitive diseases like hypertension— and increased difficulty in managing risk factors like hypertension according to clinical guidelines. [19]. Given the dual burden of FI and hypertension, evidence of negative health consequences, and paucity of evidence in the region, we aimed to explore the relationship between food insecurity and the hypertension cascade, including prevalence, awareness, and control.

## Methods

### Data source and study sample

We analyzed baseline data from the Eastern Caribbean Health Outcomes Research Network Cohort Study (ECS) for this cross-sectional study. The ECS is an ongoing longitudinal cohort study conducted across four Caribbean sites that aims to identify novel risk and protective factors for non-communicable diseases in the Eastern Caribbean region. The sites included are the U.S. Virgin Islands, Puerto Rico, Trinidad and Tobago, and Barbados. The cohort was empaneled between 2013 and 2018 across the four sites using varied sampling methods to obtain randomized samples in each site (*n* = 2,961)[20]. Eligible participants were English or Spanish-speaking community-dwelling adults 40 years of age and older who had been residents of the island for at least 10 years and intended to live on the island for the next 5 years. Participants completed a self-administered health survey including items about demographic, lifestyle, and chronic disease risk factors. They also underwent clinical examinations. Details about the ECS study methodology have been previously published [20]. ECS participants were included in the current study if they had complete blood pressure and food insecurity data at baseline. Access to ECS data can be found online at ECHORN.org [21].

### Measures

Our main exposure was food insecurity which was measured using the 9-item version of the Latin American and Caribbean Food Security Scale (ELCSA) previously validated in the Caribbean [22]. The ELCSA captured household food insecurity within the past 90 days. Response options were binary (yes/no), and one point was given for each question with a “yes” response. Responses were summed for each participant and ranged from 0 to 9. Those who scored 0 were classified as having no food insecurity, 1–6 as having mild/moderate food insecurity, and 7–9 as having severe food insecurity. The ninth ELCSA item addresses the social acceptability dimension of FI and was included as part of ELCSA’s summative score based on psychometric testing. Rasch’s modeling of the ELCSA scale in the ECS sample indicated that the full 9-item scale was the best fit [23]. The Cronbach’s Alpha for the scale was 0.90.

Our outcomes were hypertension prevalence, awareness, and control. Hypertension was defined using guidelines established by the Seventh Report of the Joint National Committee [24] and Caribbean Health Research Council [25], which were clinical guidelines used to identify hypertension during the period ECS data were collected. Hypertension was assessed using self-reported and clinical assessment data. Details about the clinical assessment processes have been previously described [26]. Participants were classified as having hypertension if they had systolic blood pressure ≥ 140 mmHg or diastolic blood pressure ≥ 90 mmHg during the clinical exam or answered “yes” to the following question, “Has a doctor or other health provider EVER told you that you have high blood pressure?” and reported taking blood pressure lowering medication. Participants who responded “no” to this question and had blood pressure <140 mmHg and diastolic blood pressure < 90 mmHg were classified as not having hypertension.

Hypertension awareness was determined by the question, “Has a doctor or other health provider EVER told you that you have high blood pressure?” Those who responded “yes” and reported taking blood pressure lowering medication were categorized and defined as “Aware.” Participants who responded “no” and had elevated systolic or diastolic blood pressure during the clinical exam were categorized as “unaware.” Hypertension control was determined among those who were aware and reported taking medication. Control was assessed using blood pressure values from the clinical exam and defined as having systolic blood pressure <140 mmHg and diastolic blood pressure <90 mmHg.

Potential confounders examined included demographic characteristics, healthcare utilization, and site. Demographic characteristics included age, sex, and educational attainment, which were measured via self-report during the ECS baseline survey. We categorized age into four groups (40–49, 50–59, 60–69, and 70+). Sex was measured on the baseline survey using the following question, “What sex were you at birth?” Educational attainment was measured using the question, “What is the highest year of school that you completed?” Responses were categorized into less than high school (or secondary school), high school graduate, some college, and college and higher. Usual source of care was used to characterize healthcare utilization and was measured using the question, “Is there one place you usually go when you need routine or non-emergent/non-emergency care (for example, regular check-up)”? Responses were categorized into three categories: none, one, or more. For analysis, usual source of care responses were categorized into two categories: none and one or more due to the low frequency of responses that indicated having two or more usual sources of care. Similar to prior study approaches [27–29], variables along the causal pathway were not included as covariates, as this may obscure the relationship between FI and hypertension prevalence, awareness, and control and result in overadjustment.

### Statistical analysis

Univariate analyses were conducted to examine the frequencies and distribution of variables. We conducted bivariate analyses using chi-square tests to examine associations between FI, demographic, and healthcare characteristics. Poisson regression was used to estimate prevalence ratios and determine unadjusted and adjusted associations between FI and hypertension prevalence, awareness, and control. Adjusted models included age, sex, educational attainment, site, and usual source of care. These covariates were selected based on evidence from prior literature [30–32]. Sex-stratified modeling was performed to determine potential differences in the impact of FI for men and women. Analyses were conducted using SAS statistical software, version 9.4 (Research Triangle Institute, Research Triangle Park).

### Ethics statement

The ECS study was approved by the Yale University Human Subjects Investigation Committee (protocol 2000026077), the Institutional Review Boards of the University of Puerto Rico Medical Sciences Campus (protocol 2290033151R001), the University of the Virgin Islands (protocol 460911–18), and the University of the West Indies Cave Hill Campus (IRB Number: 171102-A), as well as by the Ministry of Health of Trinidad and Tobago. Formal written consent was obtained from study participants. The current analysis was approved by the Data Access and Scientific Review Committee of the ECS. This study was reported according to STROBE guidelines.

## Results

**Fig 1:**
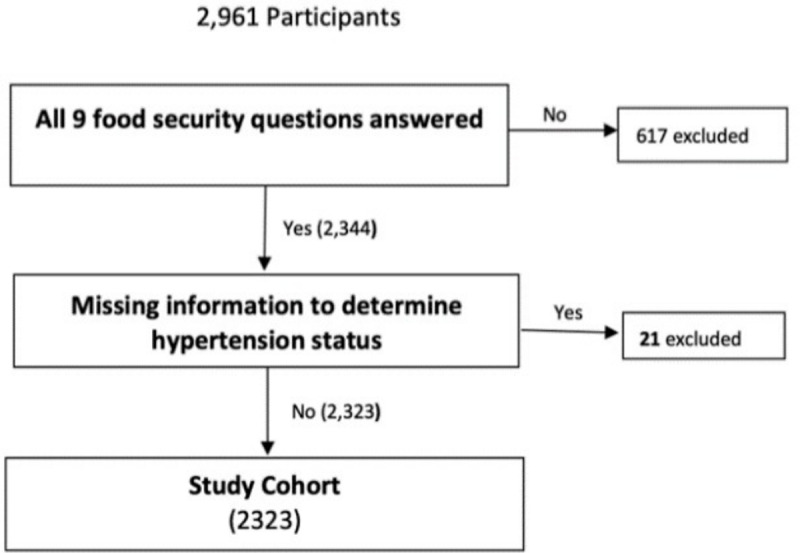
Construction of study cohort.

Our final analytic dataset included 2,323 observations. We excluded 638 participants with missing FI and blood pressure data (Fig 1). The mean age of participants was 57. Sixty-five percent of participants were women, 35 percent had less than a high school education, and most (85%) had one or more usual places where they received healthcare. The overall prevalence of FI was 28 percent among participants, and prevalence was higher in Puerto Rico and Trinidad and Tobago compared to other sites. Among those with FI, 17 percent experienced mild FI, 6 percent moderate FI, and 4 percent experienced severe FI.

Bivariate analyses showed statistically significant differences in FI by age, sex, educational attainment, and site (Table 1). Those living in households with moderate or severe FI were younger compared to those who were living in households that were food secure or had mild FI. Women and those with less than a high school education were more likely to experience mild, moderate, or severe FI compared to men and those with higher educational attainment (p < 0.05).

**Table 1:** ECS participant characteristics by food security status.

Characteristics	Total	Secure	Mild	Moderate	Severe	p-value
	2323	1683 (72.4%)	394 (17.0%)	144 (6.2%)	102 (4.4%)	
Mean age (SD)	57.3 (10.5)	58.8 (10.6)	54.1 (9.5)	51.6 (8.0)	52.9 (7.4)	<0.0001
Sex						0.0279
Men	817 (35.2)	614 (36.5)	133 (33.8)	35 (24.3)	35 (34.3)	
Women	1506 (64.8)	1069 (63.5)	261 (66.2)	109 (75.7)	67 (65.7)	
Educational attainment						<.0001
<HS	789 (34.8)	541 (32.9)	151 (39.7)	55 (39)	42 (42.0)	
HS Grad	540 (23.8)	395 (24.0)	88 (23.2)	32 (22.7)	25 (25.0)	
Some College	501 (22.1)	348 (21.2)	88 (23.2)	38 (27)	27 (27.0)	
College+	436 (19.2)	361 (21.9)	53 (13.9)	16 (11.4)	6 (6.0)	
Site						<.0001
Barbados	703 (30.3)	552 (32.8)	92 (23.4)	36 (25)	23 (22.6)	
Puerto Rico	735 (31.6)	566 (33.6)	98 (24.9)	41 (28.5)	30 (29.4)	
Trinidad and Tobago	716 (30.8)	452 (26.9)	166 (42.1)	55 (38.2)	43 (42.2)	
US Virgin Islands	169 (7.3)	113 (6.7)	38 (9.6)	12 (8.3)	6 (5.9)	
Usual source of healthcare						0.5157
None	339 (14.7)	235 (14.0)	61 (15.5)	25 (17.4)	18 (17.7)	
One or more	1974 (85.3)	1438 (86.0)	333 (84.5)	119 (82.6)	84 (82.4)	..

Table 2 presents prevalence and prevalence ratio estimates for the relationship between FI and hypertension prevalence, awareness, and control. Hypertension prevalence was 54 percent among persons with mild FI, 51 percent for moderate FI, and 52 percent for persons with severe FI. Unadjusted and adjusted model results for FI and hypertension prevalence were not statistically significant.

**Table 2:** Prevalence ratios for the association between food insecurity and hypertension outcomes.

	Hypertension Status								
	Prevalence of Hypertension			Lack of Awareness of Hypertension			Lack of Control of Hypertension		
Level of Food Insecurity	Total	Unadjusted PR (95% CI)	Adjusted PR (95% CI)	Total	Unadjusted PR (95% CI)	Adjusted PR (95% CI)	Total	Unadjusted PR (95% CI)	Adjusted PR (95% CI)
Secure (N = 1683)	991/1683 (58.9%)	1.00	1.00	346/991 (34.9%)	1.00	1.00	365/645 (56.6%)	1.00	1.00
Mild (N = 394)	211/394 (53.6%)	0.91 (0.82–1.01)	1.03 (0.93–1.14)	82/211 (38.9%)	1.11 (0.92–1.35)	0.91 (0.76–1.1)	73/129 (56.6%)	1.00 (0.85–1.18)	0.99 (0.84–1.17)
Moderate (N = 144)	73/144 (50.7%)	0.86 (0.73–1.02)	1.04 (0.89–1.22)	23/73 (31.5%)	0.9 (0.64–1.28)	0.79 (0.58–1.08)	34/50 (68%)	1.20 (0.98–1.47)	1.33 (1.09–1.64)
Severe (N = 102)	53/102 (52%)	0.88 (0.73–1.07)	1.02 (0.85–1.23)	19/53 (35.8%)	1.03 (0.71–1.49)	0.83 (0.58–1.2)	25/34 (73.5%)	1.30 (1.05–1.61)	1.30 (1.05–1.62)

* Adjusted for age, sex, education level, site, and usual source of care

Results for lack of awareness showed that 35 percent of those who lived in food-secure households and 39 percent of mild food-insecure households were unaware of their hypertension, compared to 32 percent and 36 percent among those with moderate and severe FI. Unadjusted and adjusted model results showed that FI was not significantly associated with lack of awareness of hypertension. Results for lack of hypertension control showed that 57 percent of households with mild FI, 68 percent of those with moderate FI, and 74 percent of those with severe FI did not have their hypertension controlled, compared to 57 percent among persons who were food secure. Unadjusted model results showed mild and moderate FI were not significantly associated with lack of hypertension control. However, results for severe FI were statistically significant and showed individuals in severely FI households had 30 percent higher prevalence of uncontrolled hypertension compared to those in food secure households (CI = 1.05–1.61). Adjusted model results were similar and showed moderate and severe FI was associated with 33 percent (PR = 1.33 (1.09–1.64)) greater prevalence of uncontrolled hypertension compared to those who were food secure.

Table 3 presents sex-stratified model results. Results showed that overall, women who experienced moderate or severe FI were more likely to have hypertension and lack control compared to men. Fifty-four and 58 percent of women with moderate and severe FI had hypertension, compared to 40 percent among men with moderate and severe FI. Similarly, results for lack of hypertension control showed that women with moderate (72% vs. 43%) and severe FI (81% vs. 50%) were more likely to lack control of their hypertension compared to men. Unadjusted and adjusted model results showed statistically significant positive associations between moderate and severe FI and lack of control of hypertension for women. Adjusted model results showed that women with moderate and severe FI had 40 percent higher prevalence of uncontrolled hypertension. Results for men were not statistically significant.

**Table 3:** Sex-stratified associations between food insecurity and hypertension outcomes.

	Hypertension Status								
WOMEN									
	Prevalence of Hypertension			Lack of Awareness of Hypertension			Lack of control of hypertension		
Level of Food Insecurity	Total	Unadjusted PR (95% CI)	Adjusted PR (95% CI)	Total	Unadjusted PR (95% CI)	Adjusted PR (95% CI)	Total	Unadjusted PR (95% CI)	Adjusted PR (95% CI)
Secure (N = 1069)	625/1069 (58.5%)	1.00	1.00	177/625 (28.3%)	1.00	1.00	249/448 (55.6%)	1.00	1.00
Mild (N = 261)	128/261 (49%)	0.84 (0.73–0.96)	0.99 (0.87–1.13)	42/128 (32.8%)	1.16 (0.88–1.53)	0.94 (0.71–1.25)	47/86 (54.7%)	0.98 (0.8–1.21)	0.97 (0.79–1.2)
Moderate (N = 109)	59/109 (54.1%)	0.93 (0.77–1.11)	1.15 (0.97–1.36)	16/59 (27.1%)	0.96 (0.62–1.48)	0.87 (0.58–1.3)	31/43 (72.1%)	1.3 (1.06–1.59)	1.39 (1.13–1.71)
Severe (N = 67)	39/67 (58.2%)	1.00 (0.81–1.23)	1.14 (0.94–1.38)	13/39 (33.3%)	1.18 (0.74–1.87)	0.89 (0.57–1.39)	21/26 (80.8%)	1.45 (1.18–1.78)	1.41 (1.16–1.72)
**MEN**									
	Prevalence of Hypertension			Lack of Awareness of Hypertension			Lack of control of hypertension		
Level of Food Insecurity	Total	Unadjusted PR (95% CI)	Adjusted PR (95% CI)	Total	Unadjusted PR (95% CI)	Adjusted PR (95% CI)	Total	Unadjusted PR (95% CI)	Adjusted PR (95% CI)
Secure (N = 614)	366/614 (59.6%)	1.00	1.00	169/366 (46.2%)	1.00	1.00	116/197 (58.9%)	1.00	1.00
Mild (N = 133)	83/133 (62.4%)	1.05 (0.9–1.21)	1.13 (0.97–1.31)	40/83 (48.2%)	1.04 (0.81–1.34)	0.86 (0.69–1.08)	26/43 (60.5%)	1.03 (0.79–1.34)	1.04 (0.81–1.35)
Moderate (N = 35)	14/35 (40%)	0.67 (0.44–1.01)	0.75 (0.5–1.14)	7/14 (50%)	1.08 (0.63–1.85)	0.66 (0.44–0.99)	3/7 (42.9%)	0.73 (0.31–1.73)	0.82 (0.35–1.92)
Severe (N = 35)	14/35 (40%)	0.67 (0.44–1.01)	0.75 (0.5–1.15)	6/14 (42.9%)	0.93 (0.5–1.72)	0.7 (0.37–1.32)	4/8 (50%)	0.85 (0.42–1.71)	0.93 (0.42–2.06)

* Adjusted for age, sex, education level, site, and usual source of care

## Discussion

This study aimed to determine the relationship between FI and hypertension prevalence, awareness, and control in the Eastern Caribbean. Our findings showed that FI prevalence was 28 percent and 10 percent of those households experienced moderate or severe FI. Findings also showed sex differences in experiences with FI and the relationship between FI and hypertension control. Women were more likely to experience food insecurity, and a higher proportion of women with FI had hypertension compared to men. Findings also showed a statistically significant and positive association between FI and uncontrolled hypertension among women. Results for FI and hypertension prevalence and awareness were not statistically significant. These findings further substantiate FI as a significant public health issue in the Caribbean and demonstrate its role in poor blood pressure control. In addition, our findings highlight the differential impact of FI on health outcomes among women. This study contributes to a growing evidence base on the health impacts of FI in the Caribbean, where evidence is limited yet needed to guide the creation of region-specific solutions.

Our finding of an association between FI and poor blood pressure control is novel within the Caribbean context, where few studies have examined FI and chronic diseases like hypertension. While prior studies have examined associations between FI and chronic diseases, the preponderance of evidence focuses on outcomes including diabetes, obesity, and dietary quality [33,34]. Fewer studies have examined the association between FI and hypertension or examined the role of FI in poor blood pressure control, even though it has been linked to poor dietary quality, which is known to impact hypertension control [5,35]. A recent systematic review of FI and hypertension only included 36 articles, demonstrating the paucity of evidence.

This study contributes to a growing yet mixed body of evidence on the relationship between FI and hypertension. Our results align with most prior studies, which fail to demonstrate a statistically significant association between FI and hypertension irrespective of the methods used to ascertain hypertension (e.g., self-report, clinical measures)[5,6]. While most study findings are negative, some studies that assess hypertension via self-report show statistically significant associations between FI and hypertension [5,36,37]. Evidence on the relationship between FI and hypertension awareness and control is limited. Studies that have examined FI and hypertension control association show positive associations and that individuals who experience FI are more likely to lack control of their hypertension [5,35,38,39]. Potential mechanistic pathways to explain the association between FI and poor blood pressure control include poor dietary quality, inflammation, and gut microbiome disruptions [40–42]. Social and structural factors that co-occur with FI may also explain the association. Lived experiences that include negative social and structural determinants like underemployment, unemployment, poverty, unstable housing, lack of social support, and poor quality of care can compound experiences of FI and contribute to poor hypertension control [43,44].

Findings on sex differences in FI prevalence and the differential impact of FI on blood pressure control among women are also consistent with prior studies. The consistency of these findings spans context, culture, race, and ethnicity. Several explanations have been offered. Gendered roles and inequities in earning power and household responsibilities are some explanations for the observed differences in FI between men and women [45]. In most cases, women are responsible for food purchasing and preparation. Some evidence suggests that women are more likely to sacrifice in households experiencing FI by employing strategies like reducing food consumption, reducing the diversity of foods they consume, and consuming cheaper energy-dense foods to provide for others in the household [45]. Evidence also suggests women are less likely to absorb economic and household shocks related to FI due to inequities in access to assets and resources to cope with FI [46]. Mounting evidence that shows sex differences in the relationship between FI and obesity [47–49], a risk factor for hypertension, may offer yet another explanation. Evidence shows a positive relationship between FI and obesity among women [50,51] but not men, which may complicate hypertension control among women with FI [52].

The current findings are generalizable to populations represented in the ECHORN sites and have several implications. The high burden of FI across the region and the demonstrated association with poor blood pressure control suggest an urgent need for policy and healthcare system interventions. A recent review of nutrition interventions and policies in the small island developing states, including those in the Caribbean, highlighted the paucity of nutrition policies in the region and the overall low proportion of states where policies were fully implemented [53].

### Limitations

This study has a few limitations. The cross-sectional design of our study precludes the ability to make inferences about temporality in the relationship between FI and hypertension. This may have contributed to our inability to observe a relationship between FI and hypertension prevalence and awareness. The small sample size available to examine associations between moderate and severe FI and hypertension outcomes may have precluded the ability to detect statistically significant differences despite observing significant prevalence ratios for outcomes like lack of hypertension control. Given the age of our cohort and findings that show higher FI among younger persons, our study may underestimate the overall true burden of FI in the Caribbean region. Despite these limitations, findings remain salient to efforts to reduce FI and improve blood pressure control in the region. Future studies will seek to identify food environment and healthcare system solutions that can reduce FI and improve blood pressure control among individuals experiencing FI.

## Conclusions

This study highlights the high burden of FI in the Caribbean and sex differences in the association between FI and poor hypertension control. Our findings suggest a complex interplay of social, behavioral, and clinical factors that may hinder women’s ability to achieve blood pressure control. Our study findings have implications for nutrition policies to reduce FI burden and healthcare system solutions to identify and address factors, including FI, that contribute to poor blood pressure control. Finally, studies are needed to identify and understand factors that explain sex differences in the association between FI and hypertension.

## References

[1] Food Security & Nutrition: Essential Ingredients to Build Back Better [Internet]. World Health Organization Newsroom; 2022; 18 October 2022 [cited 2024]. Available from: https://www.who.int/news-room/events/detail/2022/10/18/default-calendar/food-security-nutrition-essential-ingredients-to-build-back-better

[2] UN Report: Global hunger numbers rose to as many as 828 million in 2021. Food and Agriculture Organization of the United Nations; 2022. Available from: https://www.fao.org/newsroom/detail/un-report-global-hunger-SOFI-2022-FAO/en

[3] World Food Programme. Sharp rise in food insecurity in the Caribbean, survey finds 2022. [cited 2023 January 20]. Available from: https://www.wfp.org/news/sharp-rise-food-insecurity-caribbean-survey-finds

[4] Programme WF. Caribbean Food Security and Livelihoods Impact Survey. 2023.

[5] Beltrán S, Pharel M, Montgomery CT, López-Hinojosa IJ, Arenas DJ, DeLisser HM. Food insecurity and hypertension: a systematic review and meta-analysis. PLOS ONE. 2020;15(11):e0241628. doi: 10.1371/journal.pone.0241628 33201873 PMC7671545

[6] Oladele CR, Martinez J, Nunez-Smith M. Abstract P094: food insecurity and elevated blood pressure in the Eastern Caribbean. Circulation. 2021;143(Suppl_1):AP094-AP. doi: 10.1161/circ.143.suppl_1.P094

[7] Caribbean Public Health Agency. The Caribbean’s Blood Pressure Rates Rank Highest in the Americas. 2021 [updated 5/17/2021]. Available from: https://carpha.org/More/Media/Articles/ArticleID/467/The-Caribbeans-Blood-Pressure-Rates-Rank-Highest-in-the-Americas

[8] Figueroa J, Harris M, Duncan J, Tulloch-Reid M. Hypertension control: the caribbean needs intervention studies to learn how to do better. West Indian Med J. 2017;66(1):1–3.

[9] Mendy VL, Vargas R, Cannon-Smith G, Payton M, Enkhmaa B, Zhang L. Food insecurity and cardiovascular disease risk factors among mississippi adults. Int J Environ Res Public Health. 2018;15(9). doi: 10.3390/ijerph15092016 30223555 PMC6165024

[10] Ing CT, Clemens B, Ahn HJ, Kaholokula JKa, Hovmand PS, Seto TB, et al. Food insecurity and blood pressure in a multiethnic population. Int J Environ Res Public Health. 2023;20(13):6242. doi: 10.3390/ijerph20136242 37444090 PMC10341426

[11] Irving SM, Njai RS, Siegel PZ. Food insecurity and self-reported hypertension among Hispanic, black, and white adults in 12 states, Behavioral Risk Factor Surveillance System, 2009. Prev Chronic Dis. 2014;11:E161. doi: 10.5888/pcd11.140190 25232748 PMC4170725

[12] Afulani P, Herman D, Coleman-Jensen A, Harrison GG. Food insecurity and health outcomes among older adults: the role of cost-related medication underuse. J Nutr Gerontol Geriatr. 2015;34(3):319–42. doi: 10.1080/21551197.2015.1054575 26267444

[13] Mohammadi E, Singh SJ, McCordic C, Pittman J. Food security challenges and options in the caribbean: insights from a scoping review. Anthropocene Sci. 2022;1(1):91–108. doi: 10.1007/s44177-021-00008-8

[14] Partners CI. Food Security. [cited 2024 09 September]. Available from: https://caribimpact.com/food-security/

[15] Group WB. The Fight Against Food Insecurity in the Caribbean. World Bank; updated 28 June 2022; cited 2024 09 September. Available from: https://www.worldbank.org/en/news/feature/2022/06/28/food-insecurity-caribbean

[16] Phillips W. Food Security in the Caribbean - A Policy Perspective. 2022.

[17] Oladele CR, Colón-Ramos U, Galusha D, Tran E, Adams OP, Maharaj RG, et al. Perceptions of the local food environment and fruit and vegetable intake in the Eastern Caribbean Health Outcomes research Network (ECHORN) Cohort study. Prev Med Rep. 2022;26:101694. doi: 10.1016/j.pmedr.2022.101694 35242499 PMC8861285

[18] World Food Programme. Food Insecurity in the Caribbean continues on upward trajectory, CARICOM-WFP survey finds. World Food Programme; 2022. Available from: https://www.wfp.org/news/food-insecurity-caribbean-continues-upward-trajectory-caricom-wfp-survey-finds

[19] Grilo SA, Shallcross AJ, Ogedegbe G, Odedosu T, Levy N, Lehrer S, et al. Food insecurity and effectiveness of behavioral interventions to reduce blood pressure, New York City, 2012-2013. Preventing Chronic Dis. 2015;12:E16. doi: 10.5888/pcd12.140368 25674675 PMC4329953

[20] Thompson T, Maharaj R, Nunez M, Nazario C, Adams O, Nunez-Smith M. Non-communicable diseases III: the Eastern Caribbean health outcomes research network (ECHORN) cohort study. West Indian Med J. 2018;67:40.

[21] ECHORN. Explore ECHORN Cohort Study Data. Available from: https://www.echorn.org/explore-echorn

[22] Perez-Escamilla R, Dessalines M, Finnigan M, Pachon H, Hromi-Fiedler A, Gupta N. Household food insecurity is associated with childhood malaria in rural Haiti. J Nutr. 2009;139(11):2132–8. doi: 10.3945/jn.109.108852 . 19741201

[23] Martinez-Brockman JL, Hromi-Fiedler A, Galusha D, Oladele C, Acosta L, Adams OP, et al. Risk factors for household food insecurity in the Eastern Caribbean Health Outcomes Research Network cohort study. Front Public Health. 2023;11:1269857. doi: 10.3389/fpubh.2023.1269857 38074748 PMC10702572

[24] Chobanian AV, Bakris GL, Black HR, Cushman WC, Green LA, Izzo JL, et al. The Seventh Report of the Joint National Committee on Prevention Detection Evaluation and Treatment of High Blood Pressure the JNC 7 report. JAMA. 2003;289(19):2560–72. doi: 10.1001/jama.289.19.2560 12748199

[25] Fraser H, Hennis A, Maynard G, Poddar V, Simeon D. Managing Hypertension in Primary Care in the Caribbean. 2007.

[26] Spatz ES, Martinez-Brockman JL, Tessier-Sherman B, Mortazavi B, Roy B, Schwartz JI, et al. Phenotypes of hypertensive ambulatory blood pressure patterns: design and rationale of the ECHORN hypertension study. Ethn Dis. 2019;29(4):535–44. doi: 10.18865/ed.29.4.535 31641320 PMC6802166

[27] Dong KR, Must A, Tang AM, Stopka TJ, Beckwith CG. Food insecurity, morbidities, and substance use in adults on probation in Rhode Island. J Urban Health. 2018;95(4):564–75. doi: 10.1007/s11524-018-0290-2 30030685 PMC6095760

[28] Shiue I. People with diabetes, respiratory, liver or mental disorders, higher urinary antimony, bisphenol A, or pesticides had higher food insecurity: USA NHANES, 2005-2006. Environ Sci Pollut Res Int. 2016;23(1):198–205. doi: 10.1007/s11356-015-5677-y 26517997

[29] Weigel MM, Armijos RX, Hall YP, Ramirez Y, Orozco R. The household food insecurity and health outcomes of U.S.-Mexico border migrant and seasonal farmworkers. J Immigr Minor Health. 2007;9(3):157–69. doi: 10.1007/s10903-006-9026-6 17245658

[30] Connelly PJ, Delles C. Journal of Human Hypertension special issue on sex and gender differences in hypertension. J Hum Hypertens. 2023;37(8):587–8. doi: 10.1038/s41371-023-00847-5 37474602

[31] Lopez-Lopez JP, Cohen DD, Alarcon-Ariza N, Mogollon-Zehr M, Ney-Salazar D, Chacon-Manosalva MA, et al. Ethnic differences in the prevalence of hypertension in colombia: association with education level. Am J Hypertens. 2022;35(7):610–8. doi: 10.1093/ajh/hpac051 35437579 PMC9248921

[32] Mills KT, Stefanescu A, He J. The global epidemiology of hypertension. Nat Rev Nephrol. 2020;16(4):223–37. doi: 10.1038/s41581-019-0244-2 32024986 PMC7998524

[33] Thomas MK, Lammert LJ, Beverly EA. Food insecurity and its impact on body weight, type 2 diabetes, cardiovascular disease, and mental health. Curr Cardiovasc Risk Rep. 2021;15(9):15. doi: 10.1007/s12170-021-00679-3 34249217 PMC8255162

[34] Vaccaro JA, Huffman FG. Sex and race/ethnic disparities in food security and chronic diseases in U.S. older adults. Gerontol Geriatr Med. 2017;3:2333721417718344. doi: 10.1177/2333721417718344 28717673 PMC5502940

[35] Pérez-Escamilla R, Villalpando S, Shamah-Levy T, Méndez-Gómez Humarán I. Household food insecurity, diabetes and hypertension among Mexican adults: results from Ensanut 2012. Salud Publica Mex. 2014;56 Suppl 1:s62–70. doi: 10.21149/spm.v56s1.5167 25649455

[36] Sun Y, Liu B, Rong S, Du Y, Xu G, Snetselaar LG, et al. Food insecurity is associated with cardiovascular and all-cause mortality among adults in the United States. J Am Heart Assoc. 2020;9(19):e014629. doi: 10.1161/JAHA.119.014629 32975173 PMC7792411

[37] Leung CW, Kullgren JT, Malani PN, Singer DC, Kirch M, Solway E, et al. Food insecurity is associated with multiple chronic conditions and physical health status among older US adults. Prev Med Rep. 2020;20:101211. doi: 10.1016/j.pmedr.2020.101211 32983850 PMC7502278

[38] Liu Y, Eicher-Miller HA. Food insecurity and cardiovascular disease risk. Curr Atheroscler Rep. 2021;23(6):24. doi: 10.1007/s11883-021-00923-6 33772668 PMC8000689

[39] Hojaji E, Aghajani M, Zavoshy R, Noroozi M, Jahanihashemi H, Ezzeddin N. Household food insecurity associations with pregnancy hypertension, diabetes mellitus and infant birth anthropometric measures: a cross-sectional study of Iranian mothers. Hypertens Pregnancy. 2021;40(2):109–17. doi: 10.1080/10641955.2021.1874010 33476216

[40] Li J, Zhao F, Wang Y, Chen J, Tao J, Tian G, et al. Gut microbiota dysbiosis contributes to the development of hypertension. Microbiome. 2017;5(1):14. doi: 10.1186/s40168-016-0222-x 28143587 PMC5286796

[41] Yan Q, Gu Y, Li X, Yang W, Jia L, Chen C, et al. Alterations of the gut microbiome in hypertension. Front Cell Infect Microbiol. 2017;7:381. doi: 10.3389/fcimb.2017.00381 28884091 PMC5573791

[42] Juul F, Vaidean G, Parekh N. Ultra-processed foods and cardiovascular diseases: potential mechanisms of action. Adv Nutr. 2021;12(5):1673–80. doi: 10.1093/advances/nmab049 33942057 PMC8483964

[43] Banks AR, Bell BA, Ngendahimana D, Embaye M, Freedman DA, Chisolm DJ. Identification of factors related to food insecurity and the implications for social determinants of health screenings. BMC Public Health. 2021;21(1):1410. Epub 20210716. doi: 10.1186/s12889-021-11465-6 ; PubMed Central PMCID: PMCPMC8284017 34271906 PMC8284017

[44] Pooler JA, Hartline-Grafton H, DeBor M, Sudore RL, Seligman HK. Food insecurity: a key social determinant of health for older adults. J Am Geriatr Soc. 2019;67(3):421–4. doi: 10.1111/jgs.15736 30586154 PMC6816803

[45] Broussard NH. What explains gender differences in food insecurity? Food Policy. 2019;83:180–94. doi: 10.1016/j.foodpol.2019.01.003

[46] Seligman HK, Laraia BA, Kushel MB. Food insecurity is associated with chronic disease among low-income NHANES participants. J Nutr. 2010;140(2):304–10. doi: 10.3945/jn.109.112573 20032485 PMC2806885

[47] Hernandez DC, Reesor LM, Murillo R. Food insecurity and adult overweight/obesity: gender and race/ethnic disparities. Appetite. 2017;117:373–8. doi: 10.1016/j.appet.2017.07.010 28739148

[48] Hernandez DC, Reesor L, Murillo R. Gender disparities in the food insecurity-overweight and food insecurity-obesity paradox among low-income older adults. J Acad Nutr Diet. 2017;117(7):1087–96. doi: 10.1016/j.jand.2017.01.014 28268079

[49] Koller EC, Egede LE, Garacci E, Williams JS. Gender differences in the relationship between food insecurity and body mass index among adults in the USA. J Gen Intern Med. 2022;37(16):4202–8. doi: 10.1007/s11606-022-07714-y 35867304 PMC9708957

[50] Monroy Torres R, Castillo-Chávez AM, Ruíz-González S. [Food insecurity and its association with obesity and cardiometabolic risks in Mexican women]. Nutr Hosp. 2021;38(2):388–95. doi: 10.20960/nh.03389 33397118

[51] Schlüssel MM, Silva AA, Pérez-Escamilla R, Kac G. Household food insecurity and excess weight/obesity among Brazilian women and children: a life-course approach. Cad Saude Publica. 2013;29(2):219–26. doi: 10.1590/s0102-311x2013000200003 23459802

[52] Gooding HC, Walls CE, Richmond TK. Food insecurity and increased BMI in young adult women. Obesity (Silver Spring). 2012;20(9):1896–901. doi: 10.1038/oby.2011.233 21779092 PMC4248561

[53] Brown CR, Rocke K, Murphy MM, Hambleton IR. Interventions and policies aimed at improving nutrition in Small Island Developing States: a rapid review. Rev Panam Salud Publica. 2022;46:e33. doi: 10.26633/rpsp.2022.33 36042710 PMC9409607

